# MADRS single items differential changes among patients with melancholic and unspecified depression treated with ECT: an exploratory study

**DOI:** 10.3389/fpsyt.2024.1491451

**Published:** 2024-12-04

**Authors:** Beatriz Pozuelo Moyano, Setareh Ranjbar, Kevin Swierkosz-Lenart, Jean Pierre Schuster, Leonardo Zullo, Armin von Gunten, Pierre Vandel

**Affiliations:** ^1^ Service of Old Age Psychiatry, Department of Psychiatry, Lausanne University Hospital and University of Lausanne, Prilly, Switzerland; ^2^ Center for Research in Psychiatric Epidemiology and Psychopathology, Department of Psychiatry, Lausanne University Hospital and University of Lausanne, Prilly, Switzerland

**Keywords:** major depressive disorder, melancholic depression, unspecified depression, ECT, MADRS

## Abstract

**Introduction:**

Major depressive disorder (MDD) exhibits heterogeneity in treatment response.

**Objective:**

This exploratory analysis aims to evaluate the differential changes in individual items of the MADRS between melancholic MDD (M-MDD) and unspecified MDD (U-MDD) following electroconvulsive therapy (ECT).

**Methods:**

The study included 23 patients with unipolar MDD who received ECT. Patients were classified as M-MDD or U-MDD according to DSM-5 criteria. MADRS scores were assessed at baseline and one-month post-ECT. Differences between subtypes were analyzed using the Wilcoxon test and multiple linear regression.

**Results:**

Among 23 participants receiving ECT for MDD, 10 had M-MDD and 13 had U-MDD. Baseline MADRS items showed significantly higher scores in the M-MDD group, except for reported sadness, suicidal ideation, and concentration difficulties. Total MADRS score reduction was significantly greater in the M-MDD group. This decline was especially pronounced in M-MDD patients for specific items, including apparent sadness, inability to feel, pessimistic thoughts, sleep disturbances, reduced appetite, and concentration difficulties, after adjusting for age and sex.

**Conclusion:**

MADRS score reductions were more substantial for M-MDD than U-MDD in both total and specific items following one month of ECT. Further research with larger samples is needed to clarify MADRS response differences after ECT between melancholic and unspecified depressive subtypes.

## 1 Introduction

Major depressive disorder (MDD) has a lifetime prevalence of 15-18% (1) and exhibits diverse manifestations, clinical courses, and treatment responses, with numerous potential underlying and interconnected etiologies (2). For instance, the melancholic major depressive disorder (M-MDD) subtype is primarily characterized by anhedonia, lack of reactivity, empty mood, early morning awaking, psychomotor agitation or retardation, anorexia, and excessive guilt, and it may be associated with hypothalamic-pituitary-adrenal axis dysfunction (3–6). In addition to the interconnected etiologies underlying MDD (4, 5), temperamental traits have also been implicated in influencing the clinical presentation and treatment response of its subtypes (7).

A European multicenter study involving 1,410 individuals diagnosed with MDD, of whom 60.71% exhibited melancholic features, examined the impact of these features on the socio-demographic and clinical profiles in patients with depression. People with melancholic features had a higher body weight and exhibited higher rates of severe depressive symptoms, psychotic symptoms, suicide risk, inpatient treatment, and unemployment (8). The pharmacological profile for the M-MDD subtype appears distinct, demonstrating a lower placebo response and a more rapid response to pharmacological treatment compared to non-melancholic depression (9–13). Common treatment strategies for M-MDD patients include augmentation or combination therapies, with a preference for adjunctive treatments such as antidepressants, antipsychotics, benzodiazepines, and pregabalin (8). The unique comorbidities and prognostic characteristics of the M-MDD subtype underscore the need for tailored treatment approaches.

Electroconvulsive therapy (ECT) is a widely utilized treatment in modern psychiatry that induces a generalized convulsive seizure under general anesthesia. ECT is currently regarded as the most effective treatment for acute severe major depression (14, 15). The primary side effects are those related to general anesthesia and temporary cognitive effects, with occasional side effects including cardiac arrhythmias, confusion, increased drowsiness, urinary retention, and headache (14). There is no absolute medical contraindication for ECT (16).

MDD (both unipolar and bipolar) remains the main indication for ECT, with remission rates frequently exceeding 60% (17). Given the heterogeneity of MDD’s clinical presentation, it is appropriate to consider how different subtypes respond to ECT (18). In the case of melancholic features, a meta-analysis and systematic review examining predictive factors of response to ECT in depression analyzed seven trials reporting remission data and five trials reporting response data (19). No significant differences in response or remission were found between melancholic and non-melancholic groups (19).

While ECT is widely used and generally effective for treatment-resistant depression, there is limited evidence on the varied responses of depression subtypes (according to the DSM-5) to ECT. This gap in research is important because understanding these variations could enhance personalized treatment approaches (20).

Most of the studies assessing the specificity of MDD compare M-MDD with non-melancholic depression. However, considering the heterogeneity within depression and the presence of different subtypes (e.g., with mixed, anxious, or atypical features) (21), in this study, limited to patients who had received ECT, i.e. with M-MDD and unspecified MDD (U-MDD), rather than comparing M-MDD with all other depression types, we compare M-MDD with participants with depression who do not have characteristics of atypical or M-MDD. We believe this comparison between M-MDD and U-MDD may provide clearer insights into the specific characteristics of these two more homogenous depression subtypes.

The Montgomery-Asberg Depression Rating Scale (MADRS) (22) is a 10-item rating scale that measures the severity of depressive symptoms. MADRS is widely used in clinical and research settings as an overall measure of depressive symptoms. The traditional approach of summing symptom scores and treating depression as a single, uniform construct has been increasingly challenged by evidence highlighting the multidimensional and variable nature of major depressive disorder (23). Findings suggest that individual depressive symptoms are distinct phenomena with unique biological, functional, and risk profiles, rather than interchangeable indicators of a single underlying disorder (23). Although various factorial models have been proposed to evaluate ECT’s impact on depression, results have varied between samples, leaving implications inconclusive (24–26).

There are very few studies in the literature that examine the response to ECT on the individual items of the MADRS (27, 28). Carstens et al. analyzed the predictive value of individual MADRS items and their changes throughout ECT treatment, providing a nuanced view of ECT’s impact on specific depression symptoms (27). According to Carstens et al., each MADRS item may capture different dimensions of depression that vary among patients (27). Their findings concluded that individual MADRS items are strong predictors of ECT response, remission, and overall symptom reduction, with “apparent sadness,” “reported sadness,” and “inability to feel” items being especially predictive (27).

Identifying relevant depression subtypes and their response to ECT in treatment-resistant depression could facilitate more personalized treatment interventions. Additionally, ECT may differentially affect specific symptoms, and certain items, such as suicidal ideation, may hold greater clinical importance (29, 30). Therefore, when comparing M-MDD and U-MDD patients, we chose to use single-item scoring to examine changes in each MADRS item individually, as this approach may reveal subtle shifts otherwise obscured by aggregate scores.

In this study, we expect that the global change of MADRS scores following ECT will differ between unipolar M-MDD and U-MDD subtypes. Since each MADRS item represents a distinct symptom of depression, we also expect item-specific differences between the two subtypes after ECT. The aim of this exploratory analysis is to assess differences on the global score and individual MADRS items between M-MDD and U-MDD subtypes after one month of ECT treatment in a group of patients with unipolar depression.

## 2 Material and methods

### 2.1 Sample

Our exploratory study included a sample of 23 subjects with unipolar depression and treated with ECT. This exploratory analysis was conducted at the Interventional Unit of the Old-Age Psychiatry Service of the Lausanne University Hospital.

We reviewed medical records of patients who received ECT for M-MDD or U-MDD between January 2020 and December 2024. Baseline MADRS scores (collected prior to ECT) and 1-month MADRS scores (collected one month after initiating ECT) were obtained for analysis. Inclusion criteria required patients to be aged 18 or older, receiving ECT for their current depressive episode, diagnosed with unipolar affective disorder according to DSM-5 criteria, and having signed the general consent form for CHUV. Exclusion criteria included diagnoses of schizoaffective or bipolar disorder and any missing data essential to the study variables.

The study received approval from the Medical Ethics Committee of the Canton of Vaud (CER-VD).

### 2.2 Assessment of clinical characteristics

Demographic data, including age, sex, duration from onset of unipolar depressive disorder to ECT initiation, history of suicide attempts, presence of comorbid psychiatric disorders, and other medical conditions, were collected. MDD subtypes were determined based on DSM-5 criteria, which includes specifiers for melancholic features during the depressive episode, i.e., loss of pleasure or anhedonia and three of the following criteria: marked quality of depressed mood, depression worse in the morning, early morning awakening, psychomotor agitation or retardation, weight loss, or feelings of guilt. According to these criteria, each MDD case was classified as either M-MDD or U-MDD, meaning it did not meet criteria for atypical or melancholic features.

Depression severity at baseline and 1 month follow-up after ECT was assessed using the MADRS. The MADRS was systematically administered during the initial consultation to determine ECT indication. Baseline melancholic or unspecified features were documented from the comprehensive psychiatric evaluation conducted during this consultation. At the 1-month follow-up, the MADRS scores were either obtained from a routine consultation conducted one month after ECT initiation or reconstructed from the comprehensive psychiatric assessment conducted during the follow-up evaluation.

We also extracted a list of pharmacological treatments from medical records, documenting medications patients were receiving at the time ECT was initiated.

### 2.3 ECT procedure

ECT sessions were administered twice weekly using a Mecta machine. The initial seizure threshold was determined using the stimulus dose titration method outlined by Weiner and colleagues (31). For subsequent sessions, the dose was set at 1.5 to 2 times the seizure threshold for bilateral (BL) electrode placement and 5 times the threshold for right unilateral (RUL) electrode placement. Electrodes were positioned either right fronto-temporally for RUL or bilaterally fronto-temporally for BL. ECT was performed under general anesthesia, using etomidate and succinylcholine for muscle relaxation, with continuous monitoring of ECG, blood pressure, and pulse oximetry.

An adequate seizure was defined as one lasting at least 20 seconds by the cuff method or 25 seconds on the EEG. Dosages were adjusted throughout the treatment to ensure adequate seizure activity. All procedures were conducted by a highly trained and experienced team of psychiatrists and anesthetists.

The protocol included an initial frequency of twice-weekly sessions for a total of 12 sessions, followed by weekly sessions, with further treatment frequency and duration adjusted according to symptom progression. Participants received approximately eight ECT sessions over the first month, with the MADRS follow-up conducted at the one-month mark.

Time from the onset of depressive disorder to ECT treatment was defined as the duration from the first depressive episode to the initial ECT session, which could include multiple depressive episodes within this timeframe.

### 2.4 Statistical analysis

Descriptive statistics, including mean (SD) for continuous variables and count (percentage) for categorical variables, were used to summarize the baseline characteristics of the sample. Baseline characteristics were compared between the two MDD subtypes, M-MDD and U-MDD, using the Wilcoxon rank-sum test and Fisher’s exact test, as appropriate.

The differences in MADRS total score and its 10 individual items at baseline and 1 month after ECT treatment were compared between M-MDD and U-MDD group using Wilcoxon rank sum test as the sample size is small.

For each patient, changes in MADRS scores and its 10 individual items were calculated from baseline to the one-month follow-up after ECT treatment. Boxplots of these changes were generated for each MDD subtype group. The Wilcoxon rank-sum test was applied to assess differences in these changes between the M-MDD and U-MDD groups.

Separate multiple linear regression analyses were conducted to evaluate the differences in changes for the MADRS total score and each of its 10 subscales between the M-MDD and U-MDD groups, controlling for sex and age as covariates.

All statistical analyses were performed using the R software environment (Version 4.1.0). The significance level was set at p ≤ 0.05.

## 3 Results

A total of 23 participants met the inclusion criteria and received an acute course of ECT for MDD. The mean age of the sample was 60 years. 48% were women and 43% had a M-MDD (vs. 57% U-MDD). The mean estimated time from the onset of depressive disorder to the start of ECT treatment was 177 months; however, data on prior depressive episodes was not available in this dataset. Most patients received ECT in the BL electrode position (**Table 1**).

**Table 1:** Descriptive statistics.

Characteristics	Overall sample N = 23 n(%),mean(sd)^(a)^	M-MDD N = 10 n(%),mean(sd)	U-MDD N = 13 n(%),mean(sd)	p-value^(b)^
Sex				0.2
Male	12 (52%)	7 (70%)	5 (38%)	
Female	11 (48%)	3 (30%)	8 (62%)	
Age	60 (19)	65 (14)	57 (23)	0.6
Time onset to ECT (month)	177 (170)	198 (182)	159 (165)	0.6
Suicide attempts	3 (13%)	1 (10%)	2 (15%)	>0.9
Age onset (year)	45 (20)	50 (18)	42 (22)	0.3
Comorbidities:				
Hypertension	7 (30%)	5 (50%)	2 (15%)	0.2
Diabetes	3 (13%)	2 (20%)	1 (7.7%)	0.6
Obesity	2 (8.7%)	1 (10%)	1 (7.7%)	>0.9
Dyslipidaemia	4 (17%)	2 (20%)	2 (15%)	>0.9
History of stroke	2 (8.7%)	1 (10%)	1 (7.7%)	>0.9
History of migraine	1 (4.3%)	1 (10%)	0 (0%)	0.4
Active substance use disorder	3 (13%)	1 (10%)	2 (15%)	>0.9
Historyof substance use disorder	4 (17%)	1 (10%)	3 (23%)	0.6
History of anxiety disorder	5 (22%)	4 (40%)	1 (7.7%)	0.13
History of psychotic disorder	3 (13%)	2 (20%)	1 (7.7%)	0.6
Electrodes position				>0.9
BL	19 (83%)	8 (80%)	11 (85%)	
RUL	4 (17%)	2 (20%)	2 (15%)	

(a) Number of observation, n, and percentages (%) mean, and standard deviation(sd) are reported for categorical and continuous variables accordingly.

(b) Wilcoxon rank sum test and Fisher’s exact test were performed for continuous and categorical variables, respectively.

M-MDD, melancholic major depressive disorder; U-MDD, unspecified major depressive disorder.

At the initiation of ECT treatment, 19 out of 23 patients were on antidepressant medication, with 3 patients taking two antidepressants from different pharmacological classes simultaneously (**Supplementary Table 1**).

Patients in the M-MDD group primarily received SSRIs or SSNIs, sometimes in combination with a second antidepressant (such as trazodone or mirtazapine). In contrast, antidepressant use in the U-MDD group was more varied. At the start of ECT, 69.5% of patients were also taking a benzodiazepine, most of whom had melancholic features. The proportions of M-MDD and U-MDD patients on atypical antipsychotics were similar (**Supplementary Table 1**).

The mean baseline MADRS score was significantly higher in M-MDD patients (48) compared to U-MDD patients (35) (p < 0.001), whereas this difference is no more significant after 1 month of treatment with ECT, M-MDD patients (18) and U-MDD (21) (p=0.7) (**Supplementary Table 2**). Baseline MADRS subscores showed significantly higher scores across most items for M-MDD patients compared to U-MDD, with the exceptions of reported sadness, suicidal ideation, and concentration difficulties. However, no significant differences were found in specific item scores between M-MDD and U-MDD at the 1-month follow-up MADRS assessment (**Supplementary Table 2**).

**Figure 1:**
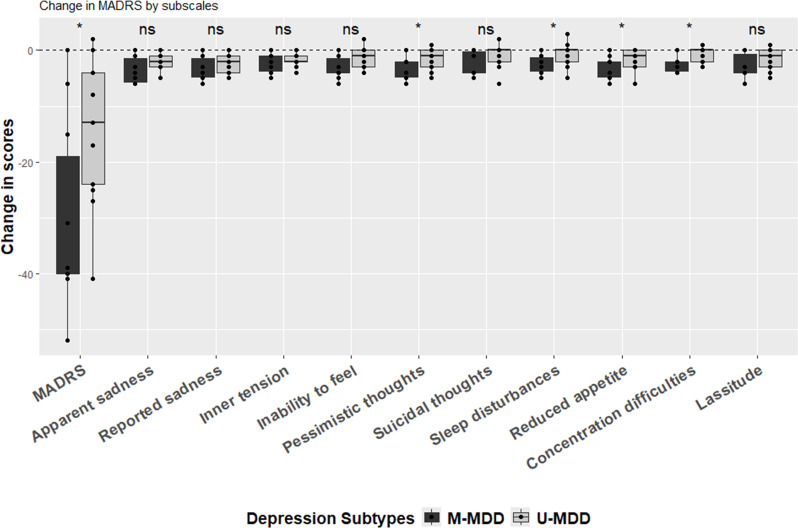
Change in MADRS (1 month after ECT-before ECT). (*) statistically significant (i.e. p<0.05) and (ns) statistically non-significant based on the unpaired two-samples Wilcoxon test.

The change in overall MADRS scores from baseline to the 1-month follow-up differed significantly (p = 0.034) between M-MDD (mean = -30, SD = 17) and U-MDD (mean = -14, SD = 13) (**Supplementary Table 3**; **Figure 1**). In the analysis of specific items, changes in pessimistic thoughts, reduced sleep, reduced appetite, and difficulty concentrating were significantly more pronounced in the M-MDD group than in the U-MDD group (**Figure 1**).

After adjusting for age and sex, the global difference in MADRS scores between baseline and 1-month follow-up for M-MDD and U-MDD groups remained significant (**Table 2**). In the specific MADRS item analysis, significant differences were observed for apparent sadness, inability to feel, pessimistic thoughts, reduced sleep, reduced appetite, and difficulty concentrating, with M-MDD patients showing a greater reduction in these symptoms (**Table 2**).

**Table 2:** Multiple linear regression (a) for Change in Overall MADRS between baseline and 1 month after ECT treatment and change in each sub-scale item.

Outcome	Estimates(β) ^(b)^	LCI	UCI	Effect-size^(c)^	P_value
**MADRS_change**	**17.45**	**5.42**	**29.48**	**1.07**	**0.007**
**Apparent sadness_change**	**1.72**	**0.05**	**3.4**	**0.83**	**0.044**
Reported sadness_change	1.38	-0.31	3.06	0.71	0.104
Inner tension_change	1.04	-0.05	2.13	0.69	0.059
**Inability to feel_change**	**1.76**	**0.28**	**3.25**	**0.89**	**0.023**
**Pessimistic thoughts_change**	**2.15**	**0.39**	**3.91**	**1.00**	**0.019**
Suicidal thoughts_change	1.81	-0.18	3.81	0.82	0.073
**Sleep disturbance_change**	**2.17**	**0.44**	**3.9**	**1.04**	**0.016**
**Reduced appetite_change**	**2.17**	**0.49**	**3.85**	**1.04**	**0.014**
**Concentration difficulties_change**	**1.61**	**0.45**	**2.76**	**1.05**	**0.009**
Lassitude_change	1.5	-0.16	2.76	0.71	0.075

(a) All Models are controlled for age and sex.

(b) β represents the coefficient for M-MMD vs U-MDD, where MD is taken as reference group.

(c) effect-size is calculated as standardized coefficient (standardized beta) from the multiple linear regression.

Bold values mean statistically significant.

## 4 Discussion

This is the first study to compare changes in M-MDD and U-MDD following ECT using a MADRS single-item model. We observed a significantly greater reduction in overall MADRS scores among participants with M-MDD compared to those with U-MDD. Specifically, focusing on individual MADRS items, we found that reductions in apparent sadness, inability to feel, pessimistic thoughts, reduced appetite, sleep disturbances, and difficulty concentrating were statistically significantly more pronounced in the M-MDD group than in the U-MDD group, after adjusting for age and sex.

Given the novel perspective of our study, direct comparisons with previous research are challenging. Previous studies assessing the effect of ECT on MDD have yielded inconclusive results regarding specific responses in depression with melancholic features, primarily due to inconsistencies in the definition of melancholia and variations in reported response and remission outcomes (32–36). The aim of this exploratory study is mainly to generate hypotheses for future prospective research.

In terms of analysis and interpretation of results, we could have opted to use a factorial model similar to that proposed by Tominaga et al. (26). Their model defines three MADRS factors: Factor 1 includes three items representing dysphoria (reported sadness, pessimistic thoughts, and suicidal thoughts); Factor 2 includes four items representing retardation (fatigue, inability to feel, apparent sadness, and difficulty concentrating); and Factor 3 includes three items representing vegetative symptoms (reduced sleep, reduced appetite, and inner tension) (26). In our study, however, we chose to analyze each item individually to capture more detailed, item-specific differences. We considered each item as potentially making an independent contribution to the overall depressive symptomatology. This approach is well supported by our findings, which show that the MADRS items demonstrating greater reductions after ECT in participants with M-MDD versus those with U-MDD span across the three factors identified by Tominaga et al. (26). It is also worth noting that certain MADRS items (e.g., difficulty concentrating) could be directly influenced by ECT-related side effects, potentially impacting the overall factor score.

Other findings are noteworthy, such as the estimated mean interval of 14.7 years between the onset of the first depressive episode and initiation of ECT in our unipolar depression population. A meta-analysis found no predictive effect of age at onset on ECT response in participants with depression (37), but we found no literature addressing the specific predictive value of this interval (time from the first depressive episode onset to ECT) on ECT outcomes.

The results focusing on the differences between M-MDD and U-MDD on the specific items of the MADRS are particularly important for several reasons. First, certain depressive symptoms are associated with increased mortality. For instance, in depressed patients, low energy, poor appetite or overeating, and lack of interest in activities have been independently linked to higher mortality from all causes and cardiovascular disease (38). Thus, based on our findings, it could be suggested that patients with M-MDD who exhibit symptoms of inability to feel, and reduced appetite might be prioritized for ECT. Clearly, this should be verified with further evidence, ideally through a prospective study with a larger sample size.

Secondly, the greater reduction in the aforementioned items in the M-MDD group following ECT suggests that for patients with severe or resistant MDD with melancholic features who experience these symptoms, ECT may be a beneficial alternative to polypharmacy. Treatment strategies for M-MDD often implies polypharmacy (8); however, pharmacotherapy alone has limited efficacy in these patients, with a response rate of approximately 40% in those with melancholic depression (10) and is associated with notable side effects (39). Introducing ECT earlier in the treatment algorithm for these patients could potentially reduce response time and minimize the side effects associated with polypharmacy.

Thirdly, residual symptoms following acute ECT treatment may predict the risk of relapse. For instance, Lambrichts et al. examined the association between individual MADRS items at the end of acute ECT and relapse at six-month follow-up in patients with late life depression (28). Their findings indicated that residual symptoms such as sleep disturbances and lassitude were significantly associated with a higher risk of relapse. This suggests that addressing these symptoms could help reduce post-ECT relapse rates in late-life depression. Although studies with larger sample sizes are needed to confirm these associations, based on the limited scientific evidence currently available, it can be hypothesized that identifying and treating M-MDD patients with ECT as a priority may be beneficial, as they could experience fewer residual symptoms after acute ECT treatment.

One possible explanation for our findings may lie in the neuroendocrine-diencephalic theory of ECT, which suggests that ECT works by correcting the neuroendocrine dysfunctions associated with M-MDD (40). M-MDD is indeed linked to hypothalamic-pituitary-adrenal (HPA) axis dysfunction, resulting in altered hormone secretion, particularly of cortisol (3–5, 40). Dysregulated cortisol levels are associated with sleep disturbances, as the HPA axis plays a key role in regulating the sleep-wake cycle, and may also contribute to appetite control issues, thereby exacerbating appetite disturbances in mood disorders. Chronic elevation of cortisol has been connected to cognitive deficits and impairments in brain function. Additionally, prolonged HPA axis activation and elevated cortisol levels may help sustain negative emotions and thoughts in individuals with mood disorders (41).

Another possible explanation could be related to the age difference between the subgroups, as the M-MDD group is on average 8 years older than the U-MDD group. Some studies suggest that age may be positively associated with ECT efficacy (19). However, after adjusting for age, the difference in MADRS score changes between the M-MDD and U-MDD groups remained significant.

Furthermore, the severity of depressive symptoms is also positively associated with response to ECT (19), and patients with melancholic features typically present with higher baseline MADRS scores (42). This was evident in our M-MDD group, which had higher baseline MADRS scores and showed a greater overall reduction in MADRS scores after ECT compared to the U-MDD group. This may help explain the observed differential response in the M-MDD group in clinical practice.

This exploratory study lays the groundwork for a prospective study to further investigate differences in MADRS outcomes following ECT in patients with late-life depression, specifically comparing those with melancholic versus unspecified features. Future prospective studies should investigate whether the differential effects of ECT on depressive symptoms in patients with M-MDD and U-MDD persist beyond the one-month treatment period used in this exploratory study, particularly as ECT session frequency decreases. Investigating specific response factors and examining the relationships between various biomarkers or temperamental traits and reductions in depressive symptoms across different depressive subtypes could yield valuable insights.

Adjusting for a list of potential confounding factors will be essential in future analyses, as these may influence the observed differences in response between subtypes; however, this will require a larger sample size. Additionally, applying a correction method, such as Bonferroni adjustment, to account for multiple comparisons will enhance the validity of the results and reduce the risk of Type I errors in the future studies where the aim extends beyond exploration.

A key hypothesis derived from the current analysis is that patients with symptoms such as apparent sadness, inability to feel, pessimistic thoughts, reduced appetite, sleep disturbances, and concentration difficulties may experience a more substantial reduction in MADRS scores following ECT. Testing this hypothesis in a larger sample and over a longer treatment period will be crucial to validate these findings and to refine personalized treatment strategies for melancholic and unspecified depression.

Moreover, future research should compare ECT with other neuromodulation techniques, such as repetitive transcranial magnetic stimulation (rTMS) and other electromagnetic therapies, as these also may show variability in response and remission rates for MDD. Using a single-item approach to MADRS in these studies could uncover subtle changes in individual symptoms that might be masked by aggregate scores, thereby allowing for a more detailed interpretation of treatment effects across neuromodulation interventions for depression.

### 4.1 Limits

One limitation in this study is that some patients received unilateral ECT, while the majority received bilateral treatment, which may impact treatment efficacy. However, the proportion of patients receiving unilateral treatment is low (17%).

Another limitation relates to the sample size, which may limit the generalizability of our findings and the ability to include all confounding factors in adjusted model, including baseline depression severity. As previously mentioned, these analyses are exploratory and intended to provide a basis for future prospective studies with a larger number of participants.

Additionally, our dataset does not include information on the history of depressive episodes between the first episode and the first ECT treatment for each participant. Although the number of previous depressive episodes is not known to be a predictor of ECT response in the general population with depression (37), investigating this association across different subtypes could yield interesting insights.

Baseline depression severity also presents a potential limitation, as patients with melancholic features often have higher initial MADRS scores, which may influence the differential response observed between subtypes. Future studies with larger samples that have overlap with respect to depression severity at baseline between M-MDD and U-MDD groups will be necessary to confirm these effects while controlling for baseline severity.

Finally, this study does not include patients with atypical features. While the original study design aimed to include melancholic, atypical, and unspecified subtypes, we did not find any patients with atypical depression who received ECT in our population according to DSM-5 criteria. This finding aligns with Husain et al. (43), which assessed remission probabilities following ECT in 453 depressed participants, of whom only 36 had atypical features (43). Interestingly, the atypical group was 2.6 times more likely to remit than the majority group with more typical features (95% CI=1.1-6.2). The reason why patients with atypical depression are rarely referred for ECT remains unclear, although this is a significant issue given that patients with atypical depression represent a substantial subgroup of MDD patients.

### 4.2 Strengths

This exploratory analysis is the first study to examine the response to each MADRS item specifically between M-MDD and U-MDD, in contrast to previous research that compared melancholic with non-melancholic patients (32–36). Another strength of this study is its naturalistic population analysis, which provides insights into how this type of intervention performs in real-world interventional psychiatry clinical practice.

## 5 Conclusion

In this exploratory study, we found a greater reduction in MADRS scores for items such as apparent sadness, inability to feel, pessimistic thoughts, reduced appetite and sleep, and difficulty concentrating in M-MDD patients compared to U-MDD patients. Although our findings should be interpreted with consideration of several limitations, they may contribute to defining a more personalized psychiatric treatment approach for severely depressed patients.

## Data Availability

The raw data supporting the conclusions of this article will be made available by the authors, without undue reservation.
