# Supplementary material for: MADRS single items differential changes among patients with melancholic and unspecified depression treated with ECT: an exploratory study
Source: Front Psychiatry. 2024 Dec 4;15:1491451. doi: 10.3389/fpsyt.2024.1491451 (PMC11652832; doi:10.3389/fpsyt.2024.1491451)
Supplement: Supplementary file 1 [file Table1.docx]

Supplementary Material

# Supplementary Tables

**Supplementary TABLE 1 :** Comedication

|  | **Overall** | **M-MDD** | **U-MDD** |
| --- | --- | --- | --- |
| Antidepressants : |  |  |  |
| SSRI | 6 | 4 | 2 |
| SSNI | 5 | 3 | 2 |
| SARI | 1 | 0 | 1 |
| SSRI and SARI | 2 | 1 | 1 |
| SSRI and TeCa | 1 | 1 | 0 |
| NDRI | 1 | 0 | 1 |
| TCA | 1 | 0 | 1 |
| Vortioxetine | 2 | 0 | 2 |
| Benzodiacepines : | 16 | 10 | 6 |
| Mood Stabilizer : |  |  |  |
| Lithium | 2 | 2 | 0 |
| Antipsychotic : |  |  |  |
| Typical | 0 | 0 | 0 |
| Atypical | 12 | 6 | 6 |
| Typical and atypical | 2 | 0 | 2 |

SSRI: selective serotonin reuptake inhibitors; SSNI: Serotonin and norepinephrine reuptake inhibitors; SARI: Serotonin antagonist and reuptake inhibitors; TeCA: Tetracyclic antidepressants; NDRI: Norepinephrine-Dopamine Reuptake Inhibitor; TCA: tricyclic antidepressant.

**Supplementary TABLE 2:** Overall score of MADRS and sub-items at baseline and after 1 month of treatment.

| **Score** | **Overall sample**  **N = 23**  **mean(sd)** | **M-MDD**  **N = 10**  **mean(sd)** | **U-MDD**  **N = 13**  **mean(sd)** | **p-value^(a)^** |
| --- | --- | --- | --- | --- |
| **Overall MADRS_baseline** | **40 (9)** | **48 (5)** | **35 (8)** | **<0.001** |
| Apparent sadness_baseline | 5 (1) | 5 (1) | 4 (1) | 0.029 |
| Reported sadness_baseline | 5 (1) | 5 (1) | 5 (1) | 0.2 |
| Inner tension_baseline | 4 (1) | 5 (0) | 4 (1) | 0.008 |
| Inability to feel_baseline | 4 (1) | 5 (1) | 3 (1) | 0.002 |
| Pessimistic thoughts_baseline | 4 (1) | 5 (1) | 4 (1) | 0.003 |
| Suicidal thoughts_baseline | 3 (2) | 4 (2) | 2 (3) | 0.092 |
| Sleep disturbances_baseline | 3 (2) | 4 (1) | 3 (2) | 0.037 |
| Reduced appetite_baseline | 3 (2) | 5 (1) | 2 (2) | 0.012 |
| Concentration difficulties_baseline | 4 (1) | 4 (1) | 4 (1) | 0.15 |
| Lassitude_baseline | 4 (1) | 5 (1) | 4 (1) | 0.002 |
| **Overall MADRS_1 month after** | **20 (12)** | **18 (15)** | **21 (11)** | **0.7** |
| Apparent sadness_1 month after | 2 (1) | 2 (2) | 2 (1) | 0.3 |
| Reported sadness_1 month after | 2 (2) | 2 (2) | 2 (1) | 0.3 |
| Inner tension_1 month after | 2 (2) | 2 (2) | 2 (2) | >0.9 |
| Inability to feel_1 month after | 2 (1) | 2 (1) | 2 (1) | >0.9 |
| Pessimistic thoughts_1 month after | 2 (2) | 2 (2) | 2 (1) | 0.7 |
| Suicidal thoughts_1 month after | 1 (2) | 1 (2) | 1 (2) | 0.9 |
| Sleep disturbances_1 month after | 2 (2) | 2 (2) | 2 (2) | >0.9 |
| Reduced appetite_1 month after | 1 (1) | 1 (2) | 1 (1) | 0.7 |
| Concentration difficulties_1 month after | 3 (2) | 2 (1) | 3 (2) | 0.10 |
| Lassitude_1 month after | 2 (1) | 2 (2) | 2 (1) | 0.8 |

(a) Wilcoxon rank sum test.

**Supplementary TABLE 3:** Univariate analysis of difference in MADRS change and its sub-items.

| **Outcome** | **Overall sample**  **N = 23**  **mean(sd)** | **M-MDD**  **N = 10**  **mean(sd)** | **U-MDD**  **N = 13**  **mean(sd)** | **p-value^(a)^** |
| --- | --- | --- | --- | --- |
| **MADRS_change** | **-21 (16)** | **-30 (17)** | **-14 (13)** | **0.034** |
| Apparent sadness_change | -3 (2) | -4 (2) | -2 (2) | 0.10 |
| Reported sadness_change | -3 (2) | -3 (2) | -2 (2) | 0.2 |
| Inner tension_change | -2 (2) | -2 (2) | -2 (1) | 0.4 |
| Inability to feel_change | -2 (2) | -3 (2) | -1 (2) | 0.058 |
| Pessimistic thoughts_change | -2 (2) | -4 (2) | -2 (2) | 0.034 |
| Suicidal thoughts_change | -2 (2) | -3 (2) | -1 (2) | 0.064 |
| Sleep disturbances_change | -1 (2) | -2 (2) | -1 (2) | 0.047 |
| Reduced appetite_change | -2 (2) | -3 (2) | -2 (2) | 0.044 |
| Concentration difficulties_change | -1 (2) | -2 (2) | -1 (1) | 0.015 |
| Lassitude_change | -2 (2) | -3 (2) | -1 (2) | 0.2 |

(a) Wilcoxon rank sum test.
